# Safety and tolerability of autologous bone marrow mesenchymal stromal cells in ADPKD patients

**DOI:** 10.1186/s13287-017-0557-7

**Published:** 2017-05-23

**Authors:** Atieh Makhlough, Soroosh Shekarchian, Reza Moghadasali, Behzad Einollahi, Seyedeh Esmat Hosseini, Neda Jaroughi, Tina Bolurieh, Hossein Baharvand, Nasser Aghdami

**Affiliations:** 1 Department of Nephrology, Molecular and Cell Biology Research Center, Sari University of Medical Sciences, Sari, Iran; 2 Department of Regenerative Biomedicine, Cell Science Research Center, Royan Institute for Stem Cell Biology and Technology, ACECR, Tehran, Iran; 3 Department of Stem Cells and Developmental Biology, Cell Science Research Center, Royan Institute for Stem Cell Biology and Technology, ACECR, Tehran, Iran; 4 Nephrology and Urology Research Center, Baqiyatallah University of Medical Sciences, Baqiyatallah Hospital, Tehran, Iran

**Keywords:** Autosomal dominant polycystic kidney disease, Bone marrow mesenchymal stromal cells, Chronic kidney disease

## Abstract

**Background:**

Autosomal dominant polycystic kidney disease (ADPKD) is a genetic ciliopathy disease characterized by progressive formation and enlargement of cysts in multiple organs. The kidneys are particularly affected and patients may eventually develop end-stage renal disease (ESRD). We hypothesize that bone marrow mesenchymal stromal cells (BMMSCs) are renotropic and may improve kidney function via anti-apoptotic, anti-fibrotic, and anti-inflammatory effects. In this study, we aim to assess the safety and tolerability of a BMMSC infusion in ADPKD patients.

**Methods:**

We performed a single-arm phase I clinical trial with a 12-month follow-up. This study enrolled six eligible ADPKD patients with an estimated glomerular filtration rate (eGFR) of 25–60 ml/min/1.73 m^2^. Patients received autologous cultured BMMSCs (2 × 10^6^ cells/kg) through the cubital vein according to our infusion protocol. We investigated safety issues and kidney function during the follow-up visits, and compared the findings to baseline and 1 year prior to the intervention.

**Results:**

There were no patients lost to follow-up. We observed no cell-related adverse events (AE) and serious adverse events (SAE) after 12 months of follow-up. The mean eGFR value of 33.8 ± 5.3 ml/min/1.73 m^2^ 1 year before cell infusion declined to 26.7 ± 3.1 ml/min/1.73 m^2^ at baseline (*P* = 0.03) and 25.8 ± 6.2 ml/min/1.73 m^2^ at the 12-month follow-up visit (*P* = 0.62). The mean serum creatinine (SCr) level of 2 ± 0.3 mg/dl 1 year before the infusion increased to 2.5 ± 0.4 mg/dl at baseline (*P* = 0.04) and 2.5 ± 0.6 mg/dl at the 12-month follow-up (*P* = 0.96). This indicated significant changes between the differences of these two periods (12 months before infusion to baseline, and 12 months after infusion to baseline) in SCr (*P* = 0.05), but not eGFR (*P* = 0.09).

**Conclusions:**

This trial demonstrated the safety and tolerability of an intravenous transplantation of autologous BMMSCs. BMMSC efficacy in ADPKD patients should be investigated in a randomized placebo-controlled trial with a larger population, which we intend to perform.

**Trial registration:**

ClinicalTrials.gov, NCT02166489. Registered on June 14, 2014.

**Electronic supplementary material:**

The online version of this article (doi:10.1186/s13287-017-0557-7) contains supplementary material, which is available to authorized users.

## Background

Autosomal dominant polycystic kidney disease (ADPKD) is a genetic ciliopathy disease characterized by progressive formation and enlargement of cysts in multiple organs. The kidneys are particularly affected and patients may eventually progress to end-stage renal disease (ESRD). ADPKD is the most common genetic renal disease [1] that affects approximately 4 to 7 million individuals [2]. Mutations in polycystins 1 and 2 impair homeostasis and intracellular calcium signaling, which subsequently cause cyst formation, inflammation, and fibrosis in the kidneys [3, 4].

Despite recent advances in the identification of ADPKD pathogenesis, current treatments have been unable to completely cure or control disease progression. Tolvaptan, a vasopressin II receptor antagonist (V2RA), is the only drug that has passed a phase III clinical trial. Though it has the ability to reduce progression of height-adjusted total kidney volume (htTKV), it is associated with numerous adverse effects [5].

Transplantation of mesenchymal stromal cells (MSCs) is a recent approach to treat renal disease [6, 7]. MSCs are plastic-adherent cells that must at least express CD73, CD90, and CD105 and lack expression of CD45, CD34, CD14 and human leukocyte antigen (HLA)-DR. They have the potential for in vitro differentiation to osteoblasts, adipocytes, and chondroblasts [8]. Several studies report their ability to treat renal diseases by different mechanisms [9]. Anti-apoptotic [10], anti-fibrotic [11], and anti-inflammatory [12] capabilities of MSCs [7] provide the basis for their administration to control the progression of polycystic kidney disease (PKD). Franchi et al. have reported that transplanted MSCs restored kidney function in a PKD model and improved damaged vasculature of these kidneys [13]. Previously we showed the promising efficacy of transplanted MSCs in experimental models of chronic kidney disease (CKD) and acute kidney injury (AKI) [14–16]. We had indicated that MSC-derived conditioned medium induced renal tubular cell regeneration after nephrotoxicity induction [14], then we showed the bone marrow MSC (BMMSC) lowered serum creatinine (SCr) and urea levels and accelerated regulatory T cells (Tregs) in a monkey AKI model [15]. In a recent study, our results indicated that MSCs resulted in decreased CKD progression in a monkey model [16]. Carvalhosa et al. reported that cells lining cysts in ADPKD consisted of polycystic CD133+ progenitor [17]. Our unpublished data showed that in vitro co-culture of MSCs and tubular CD133+ progenitor cells from ADPKD patients resulted in reduced cyst formation and proliferation potential.

We hypothesize that MSCs are renotropic. Therefore, this study aims to assess the safety and tolerability of MSC infusion in ADPKD patients.

## Methods

### Study design

We performed a single-arm phase I clinical trial at one trial center with a 12-month follow-up period. We investigated safety issues and kidney function during the follow-up visits, and compared the findings to baseline and 1 year prior to intervention. Table 1 shows the study assessment schedule. We evaluated 27 ADPKD patients for potential participation in this trial. We had diagnosed all these patients according to unified imaging diagnostic criteria [18]. Patients were under our observation for at least 1 year prior to study enrollment. Their blood pressures (BP) were well controlled. From these potential participants, we recruited six eligible ADPKD patients who provided written informed consent for study participation. This study enrolled patients from June 2014 to January 2015. All patients have been on a special diet that included decreased salt, protein, and caffeine consumption, and high water intake, recommended by our nutritionist. We strongly recommended that patients keep track and not change their medications, supplements, or make any lifestyle changes. This trial was registered with clinical trial number NCT02166489.

**Table 1:** Assessment schedule

	SV	BV1	V1	BV2	V2	V3	V4	V5	V6	V7
Day	-56	-49	-42	0	1	30	90	180	270	360
Time window	NA	±3d	±7d	±1d	±1d	±7d	±7d	±7d	±7d	±7d
Informed consent	x									
Physical examination	x	x		x		x	x	x	x	x
Vital signs	x	x	x	x		x	x	x	x	x
Medical history	x									
Laboratory assessments	x			x		x	x	x	x	x
CBC				x		x	x	x	x	x
BG				x		x	x	x	x	x
NA, K, Ca, P				x		x	x	x	x	x
BUN				x		x	x	x	x	x
Uric acid				x		x	x	x	x	x
Serum creatinine	x			x		x	x	x	x	x
eGFR	x			x		x	x	x	x	x
Albumin				x				x		x
TG, Chol, LDL,HDL				x		x	x	x	x	x
FBS	x			x		x	x	x	x	x
HbA1c	x									x
TSH, PTH				x						x
ESR, CRP				x		x	x	x	x	x
ALT, ALK-p, AST				x		x	x	x	x	x
Dipstick proteinuria				x		x	x	x	x	x
HIV 1,2, HCV, HBV, HTLV	x									
Eligibility criteria assessment	x			x						
Kidney sonography		x								x
Kidney DTPA scan		x								x
BM harvesting			x							
MSC infusion					x					
Follow-up visit						x	x	x	x	x
AE assessment ^a^					x	x	x	x	x	x
SAE assessment ^a^					x	x	x	x	x	x

*SV* screen visit, *BV1* baseline visit before bone marrow aspiration (BMA), *BV2* baseline visit before infusion day, *V* visit, *d* day, *CBC* complete blood count, *BUN* blood urea nitrogen, *eGFR* estimated glomerular filtration rate, *TG* triglycerides, *Chol* cholesterol, *LDL* low-density lipoprotein, *HDL* high-density lipoprotein, *FBS* fasting blood sugar, *HbA1c* hemoglobin A1c, *TSH* thyroid-stimulating hormone, *PTH* parathyroid hormone, *ESR* erythrocyte sedimentation rate, *CRP* C-reactive protein, *ALT* alanine aminotransferase, *AlK-p* alkaline phosphatase, *AST* aspartate aminotransferase, *HIV* human immunodeficiency virus, *HCV* hepatitis C virus, *HBV* hepatitis B virus, *HTLV* human T-lymphotropic virus, *DTPA* diethylenetriaminepentaacetic acid, *BM* bone marrow, *MSC* mesenchymal stromal cell, *AE* adverse event, *SAE* serious adverse event

^a^AE and SAE were assessed during the follow-up visit and when reported by patients

We evaluated both safety and tolerability of the cell infusion according to physical examination, adverse event (AE) assessments, and laboratory changes. Potential efficacy included changes in kidney length (KL) measured by ultrasound imaging, estimated glomerular filtration rate (eGFR) by the modification of diet in renal disease (MDRD) study formula, and glomerular filtration rate (GFR) by diethylenetriaminepentaacetic acid (DTPA) scan.

We abstracted data that pertained to the “1 year prior to infusion” point from patients’ medical files in the clinic. We scheduled patient follow-up visits for clinical and laboratory assessments at specified intervals of 1, 3, 6, 9, and 12 months after the cell infusion.

### Enrollment criteria

Inclusion criteria were: (1) both genders; (2) ADPKD confirmed by sonography imaging or genetic testing; (3) age 18 to 60 years; (4) eGFR 25–60 mL/min/1.73 m^2^; (5) ability to understand and willingness to sign a consent form. Exclusion criteria consisted of: (1) pregnancy or breastfeeding; (2) positive history of associated cardiovascular disease; (3) diabetes that required medical intervention; (4) other systemic diseases that involved the kidneys such as cancers, autoimmune diseases, blood diseases, and liver diseases; (5) hospitalization due to illness 2 months prior to study entry; (6) life expectancy less than 2 years; and (7) allergies to the cell culture ingredients.

### Primary and secondary endpoints

Primary endpoints consisted of the numbers, type, and severity of AEs related to the cell infusion. We recorded AEs and serious adverse events (SAE) during follow-up visits and whenever patients reported any symptoms. We recorded the type and grade of AEs according to the Common Terminology Criteria for Adverse Events (CTCAE) version 4.0. We sent these reports to the Data Safety Monitoring Board (DSMB) of the study. Secondary endpoints consisted of changes in eGFR (as an existing surrogate endpoint) [19] from baseline to 12 months after the cell infusion.

### Kidney ultrasound imaging and kidney DTPA scan

Additional file 1 lists this information.

### Bone marrow aspiration (BMA) procedure, cell preparation, and culture

Once the patients’ laboratory viral tests were normal (Additional file 1: Table S1), an oncologist performed a bone marrow aspiration (BMA) under local anesthesia (2% lidocaine solution) and an intravenous sedation that consisted of 0.1 mg/kg midazolam and 25–50 mg intravenous fentanyl. The BMA was obtained from the posterior superior iliac crest with the patient placed in the lateral decubitus position. Samples were placed inside a cold box and sent to the clean room. The oncologist discharged all patients 4 h after the procedure. We telephoned patients 2 days later to ask about any complications related to the BMA procedure. None of the patients reported any complications. Bone marrow (BM) mononuclear cells were plated at 1 × 10^6^ cells/cm^2^ in a 150-cm^2^ culture flask with alpha modified Eagle medium (α-MEM; 22571-020, Gibco BRL, Karlsruhe, Germany) supplemented with 100 IU penicillin and 100 IU streptomycin (15070-063, Gibco), 10% Hyclone defined fetal bovine serum (FBS; SH30070.03, Thermo Fisher Scientific, Waltham, MA, USA), and 1% L-glutamine (25030-024, Gibco). Phase-contrast microscopy of the BMMSC cultures demonstrated a low heterogeneous population that predominately consisted of long, spindle-shaped cells. For each patient, we characterized the cells for immunophenotypic properties that included surface marker expressions for MSCs - phycoerythrin (PE)-conjugated CD105, CD44, CD73 (Becton Dickinson, Franklin Lakes, NJ, USA) and fluorescein isothiocyanate (FITC)-conjugated CD90 (Dako, Glostrup, Denmark) along with those that were negative for hematopoietic stem cell (HSC) surface markers PE-conjugated CD34 and FITC-conjugated CD45. Negative isotype controls included nonspecific mouse IgG1-FITC/IgG1-PE and IgG2a-FITC that were substituted for the primary antibody. All samples were analyzed by flow cytometry (BD FACS Caliber, BD Biosciences, San Jose, CA, USA) and win-MDI2.9 software. Additional file 2: Figure S1 show MSC characterization of the enrolled patients. The cells were tested for possible microbial contamination prior to infusion. We used quality control tests (QCT) in accordance with the recommendations for cell and tissue therapy promotion and validation tests of the Iranian Health Ministry Pharmacopoeia Commission and the Department of Health and Human Services, Food and Drug Organization (FDO). Tests included the sample microbial test, limulus amebocyte lysate (LAL) gel clot assay, mycoplasma detection, and karyotype analysis.

Passages 1 to 3 of the cultured BMMSCs were washed with phosphate-buffered saline and trypsinized with trypsin/EDTA (0.05%; 25300-062, Gibco). The cells were diluted in 10 ml of normal saline and loaded into 10 ml sterile syringes. For each patient, we prepared approximately 1–2 × 10^6^ cells/kg, which were delivered to the institute’s operating room in a cold box at a temperature of approximately 4 °C.

### Cell infusion

We infused autologous cultured MSCs (1–2 × 10^6^/kg) through the cubital vein according to our infusion protocol.

### Statistical analysis

Data are written as mean ± standard deviation (SD). Following normal distribution of parametric data, we used the paired *t* test to compare variables at two time points (baseline and 12 months after cell infusion). We compared variables at three time points (12 months before infusion, baseline, and 12 months after infusion) by the repeated measures ANOVA test. A two-sided *P* value of <0.05 was considered statistically significant. We used SPSS for statistical analyses (Version 16, SPSS Inc., Chicago, IL, USA).

## Results

### Patient characteristics

All six patients completed the trial. No patients were lost to follow-up. Table 2 lists the patients’ characteristics and demographic data at the time of enrollment. Additional file 1: Table S1 shows the cell parameters. All patients’ BP was under tight control prior to enrollment. Patients’ antihypertensive medications did not change during the trial. There were five patients with children. None of these patients underwent any procedures to prevent the transmission of the disease gene to their children. One patient has not attempted to have any children. None experienced external renal complications due to PKD prior to enrollment. We did not observe any external renal complications from PKD during the 12 months of follow-up. Although one patient had autosomal dominant polycystic liver disease (ADPLD), we did not observe any changes in the size of her liver by ultrasonography at the 12-month follow-up.

**Table 2:** Patient characteristics and demographic data at the time of enrollment

Patient number	1	2	3	4	5	6
Sex (m/f)	F	M	M	M	F	F
Age (years)	45	37	39	28	51	54
Race	Caucasian	Caucasian	Caucasian	Caucasian	Caucasian	Caucasian
Educational level	Graduate studies	Completed high school	Graduate studies	Graduate studies	Completed high school	Completed high school
Marital status	Married	Married	Married	Married	Married	Married
Number of children	2	1	2	0	2	2
Employment	Full-time employment	Full-time employment	Part-time employment	Full-time employment	Retired	Homemaker
Diagnosis due to	Incidental imaging	Screening	Screening	Screening	Screening	Screening
BMI (kg/m^2^)	29.38	26.45	26.18	35.51	24.34	23.41
Blood group	O+	A-	A+	O+	B+	B+
BP sitting position (mm Hg)	135/80	115/79	110/70	131/70	133/73	127/83
eGFR (ml/min/1.73 m^2^)	25	32	25	25	25	29
GFR DTPA SCAN	23	41.6	24	28	26	32.83
Serum creatinine (mg/dl)	2.3	2.3	2.8	3.0	2.5	1.9
Dipstick proteinuria^a^	Trace	Neg	Trace	Neg	Neg	+
Previous symptoms or complication of ADPKD (yes/no)						
Cyst infection	N	N	N	N	N	N
Urinary infection	Y	N	N	Y	N	N
Flank pain	N	N	N	Y	Y	N
Macrohematuria	N	N	N	Y	Y	N
Other	N	N	N	N	N	N
Past medical history (yes/no)						
DM	N	N	N	N	N	N
HTN	Y	Y	Y	Y	Y	Y
ADPLD	N	N	N	N	N	Y
Other (name)	L4, L5 laminectomy, kidney stone	Varicocelectomy	Low back pain, kidney stone	Incarcerated spinal disc, canal stenosis, kidney stone, fatty liver grade I	Hypothyroidism, kidney stone, C/S surgery	Hysterectomy
Diagnosis of HTN, age	40	32	19	22	36	44
Duration of CKD at enrollment (estimation by month)	62	132	36	14	120	120
Smoking history (yes/no)	N	N	N	N	N	N
Alcohol consumption history (yes/no)	N	N	N	N	N	N
Family history of ADPKD (yes/no)	N	Y	Y	Y	Y	Y
Under controlled diet (yes/no)^b^	Y	Y	Y	Y	Y	Y
Drug list (name/dose)	Metoral 50 mg/daily calcitrol I daily PD-poetin each 10 days	Gemfibrozil 300 mg/daily losartan 25 mg/daily amlodipine 5 mg/daily allopurinol 100 mg/daily calcium I daily	Enalapril 5 mg/daily losartan 25 mg/daily	Losartan 25 mg/daily atenolol 50 mg/daily diltiazem 60 mg/daily prazocin 5 mg/daily allopurinol 100 mg/daily, nephrovit I daily, vit D_3_ I biweekly	Losartan 25 mg ½ daily atorvastatin 20 mg/daily allopurinol 100 mg ½ daily levothyroxine ½ daily calcitrol in 5 days of week	Losartan 50 mg bid amilodipine 50 mg/daily allopurinol 100 mg/daily

*BMI* body mass index, *BP* blood pressure, *eGFR* estimated glomerular filtration rate, *DTPA* diethylenetriaminepentaacetic acid, *ADPKD* autosomal dominant polycystic kidney disease, *DM* diabetes mellitus, *HTN* hypertension, *ADPLD* autosomal dominant polycystic liver disease, *CKD* chronic kidney disease, *NA* not applicable, *N* no, *Y* yes

^a ^Neg: 0 mg/dl; Trace: 15–30 mg/dl; +: 30–100 mg/dl; ++: 100–300 mg/d; +++: 300–1000 mg/dl; ++++: >1000 mg/dl

^b ^Under controlled diet is defined as: no: on normal diet; yes: patients have been under the recommendation of nutritionist by taking low protein, low salt diet, with low caffeine and high intake of water

### Safety and tolerability

We discharged all patients 2 h after the cell infusion and observed no AEs during this time. The bone marrow puncture site healed within 72 h and the intravenous infusion site healed within 24 h without any related complications. In total, we recorded 1 SAE and 37 AEs during the 12-month follow-up in all patients (Additional file 1: Table S2).

We sent the related AE reports to the DSMB of the trial as scheduled. The DSMB raised the issue that vomiting and dizziness AEs occurred shortly after the DTPA kidney scan in the majority of patients at the baseline and follow-up visits. They recommended that we cancel the DTPA kidney scans for the rest of the trial.

According to DSMB comments, all other AEs were unrelated to the intervention and probably attributed to patients’ underlying disease progression or other medical conditions. All AEs resolved spontaneously or by the use of over-the-counter medications.

One patient experienced an SAE. Patient number 3 who had a positive history for low back pain took a single dose of a celecoxib tablet (200 mg) and one acetaminophen tablet (500 mg) due to severe back pain 3 weeks after his cell infusion. His blood urea nitrogen (BUN) and SCr levels elevated 24 h after celecoxib and acetaminophen. We advised him against using these types of medications. Instead, we prescribed physiotherapy for pain relief. After 1 week, his BUN and SCr values returned to previous levels. We referred him to the orthopedic surgeon who performed a lumbosacral magnetic resonance imaging (MRI) scan without contrast. According to the MRI report, the patient had L4/L5 disc demyelination and a central disc protrusion with significant canal and left foraminal stenosis. He underwent surgery 1 week later. After surgery, his pain reduced and cleared completely within 10 days.

### Primary endpoint

We did not detect any SAEs or AEs related to the cell infusion in any of the patients during the 12-month follow-up. The results showed no significant differences in laboratory parameters, which we evaluated for safety issues after 1, 3, 6, 9 (Additional file 1: Table S3) and 12 months compared to baseline (Table 3).

**Table 3:** Laboratory parameters at baseline and 12-month follow-up

Patient parameters	Normal range	Baseline (Mean ± SD)	12-month (Mean ± SD)	Difference (Mean ± SD)	*P* value (Paired *t* test)
Leukocytes (/ul)	4000–10000	5700 ± 885.4	6468.3 ± 2153.2	768.3 ± 1406.9	0.24
Hemoglobin (g/dl)	12–16	12.8 ± 1.7	13 ± 1.2	0.2 ± 0.8	0.55
HCT (%)	38–47	37.4 ± 3.8	39.5 ± 3.4	2.1 ± 2.9	0.15
MCV (fl)	80–96	88.8 ± 9.4	89.5 ± 3.3	0.7 ± 7.8	0.82
Platelets (*10^3^/ul)	150–450	211.7 ± 56.8	187.3 ± 42	-24.3 ± 46	0.25
FBS (mg/dl)	60–105	96.8 ± 10	87.4 ± 11.9	-6.2 ± 7.2	0.13
HbA1c (%)	4–6	5.2 ± 0.3	5.1 ± 0.40	0 ± 0.1	0.3
Sodium (mEq/l)	134–148	141.8 ± 1.9	141.3 ± 2	-0.5 ± 1.2	0.36
Potassium (mEq/l)	3.5–5.5	4.4 ± 0.5	4.3 ± 0.5	-0.1 ± 0.4	0.41
Calcium (mg/dl)	8.6–10	9.3 ± 0.6	9.4 ± 0.3	0 ± 0.8	0.79
Phosphorus (mg/dl)	2.6–4.5	3.7 ± 0.6	3.7 ± 0.5	0 ± 0.5	0.89
TSH (MIU/ml)	0.2–5.0	2.3 ± 1.1	2.4 ± 1.3	0.1 ± 1.7	0.86
PTH (pg/ml)	15–65	140.3 ± 71	112 ± 48.3	-29.2 ± 55.2	0.25
ESR 1 h (mm/h)	3–20	22.2 ± 18.8	15.5 ± 10.3	-6.7 ± 9.9	0.16
CRP (mg/l)	0–10	5.1 ± 8.7	0.3 ± 0.9	-4.7 ± 8.5	0.23
Albumin (g/dl)	3.5–5.2	4.3 ± 0.3	4.4 ± 0.5	0.1 ± 0.6	0.68
Uric acid (mg/l)	2.6–6	6.5 ± 2.1	7.2 ± 1.3	0.7 ± 1.7	0.38
ALT(U/l)	0–40	24.3 ± 9.8	19.3 ± 6.6	-5.0 ± 11.5	0.34
AST (U/l)	0–31	29.7 ± 18.5	20.5 ± 5.8	-9.1 ± 17.4	0.25
Alkaline phosphatase (U/l)	0–240	231.2 ± 62.5	155.8 ± 53.8	-75.3 ± 83.9	0.07
Total cholesterol (mg/dl)	<200 desirable	177 ± 44.2	172.5 ± 42	-4.5 ± 23.7	0.66
Triglycerides (mg/dl)	<200 desirable	191.7 ± 150.7	192.5 ± 108.2	0.8 ± 62.8	0.97
LDL cholesterol (mg/dl)	<100 low risk	99.5 ± 34	96 ± 30.2	-3.5 ± 18.8	0.67
HDL cholesterol (mg/dl)	>55 no risk	39.2 ± 11	41.3 ± 12.3	2.2 ± 9.8	0.61
Dipstick proteinuria^a^	Negative	1.7 ± 0.8	1.3 ± 0.5	-0.3 ± 1	0.46

*ul* microliter, *g/dl* grams per deciliter, *HCT* hematocrit, *MCV* mean corpuscular volume, *fl* femtoliters, *FBS* fasting blood sugar, *HbA1c* hemoglobin A1c, *mg/dl* milligram per deciliter, *mEq/l* milliequivalents per liter, TSH thyroid-stimulating hormone, *MIU/ml* milli-international units per milliliter, *PTH* parathyroid hormone, *pg/ml* pictogram per milliliter, *ESR* erythrocyte sedimentation rate, *mm/h* millimeters per hour, *CRP* C-reactive protein, *mg/l* milligram per liter, *ALT* alanine aminotransferase, *U/lit* units per liter, *AST* aspartate aminotransferase, *LDL* low-density lipoprotein, *HDL* high-density lipoprotein

^a ^Negative or 1 = 0 mg/dl; 2 = 15–30 mg/dl; 3 = 30–100 mg/dl; 4 = 100–300 mg/d; 5 = 300–1000 mg/dl; 6 > 1000 mg/dl

### Secondary endpoint

The single-dose autologous MSC infusion did not induce any significant changes in eGFR, nor reductions in SCr at 12 months compared to baseline in all patients (Table 4).

**Table 4:** Changes in renal function parameters and blood pressure (BP) from 1 year before mesenchymal stromal cell (MSC) infusion, baseline and the 12-month follow up

Patient parameters	Normal range	-12	Baseline	12-month	*P* value (RMA)	Dif 1 (BV-1yp)	*P* value Dif 1	Dif 2 (12 m-BV)	*P* value Dif 2	Dif 3 (dif2- dif1)	*P* valve Dif 3
SBP (mmHg)	<120	123.8 ± 9.6	125.2 ± 10.3	126.8 ± 5.7	0.55	1.3 ± 6.8	0.65	1.6 ± 5.0	0.46	NA	NA
DBP (mmHg)	60-80	77.7 ± 3.5	75.8 ± 5.6	78.8 ± 3.3	0.43	-1.8 ± 4.5	0.36	3 ± 6	0.27	NA	NA
BUN (mg/dl)	7–20.6	28.5 ± 8.6	27.8 ± 10.5	29.5 ± 7.1	0.92	-0.6 ± 12.7	0.90	1.7 ± 4.3	0.38	NA	NA
SCr (mg/dl)	0.4–1.4	2 ± 0.3	2.5 ± 0.4	2.5 ± 0.6	0.02^b^	0.5 ± 0.4	0.04^b^	0 ± 0.3	0.96	0.5 ± 0.5	0.05^b^
eGFR^a^	90–120	33.8 ± 5.3	26.7 ± 3.1	25.8 ± 6.2	0.01^b^	-7.2 ± 6	0.03^b^	-0.8 ± 3.9	0.62	-6.3 ± 7.5	0.09
Rt KL (cm)	10–12	NA	19.1 ± 2	19.5 ± 2	NA	NA	NA	0.4 ± 0.6	0.13	NA	NA
Lt KL (cm)	10–12	NA	18.3 ± 2.4	18.7 ± 2.6	NA	NA	NA	0.4 ± 0.4	0.06	NA	NA

*BV* baseline visit, *1yp* 1 year prior to intervention, *12 m* 12 month after baseline, *RMA* repeated measures ANOVA, *Dif* difference, *SPB* systolic blood pressure, *mmHg* millimeter of mercury, *NA* not applicable, *DBP* diastolic blood pressure, *BUN* blood urea nitrogen, *mg/dl* milligram per deciliter *SCr* serum creatinine, *eGFR* estimated glomerular filtration rate, *Rt* right, *KL* kidney length, *Lt* left

^a ^MDRD study formula (ml/min/1.73 m^2^)

^b ^Significant

#### eGFR, sCr, and blood pressure (BP)

**Fig. 1:**
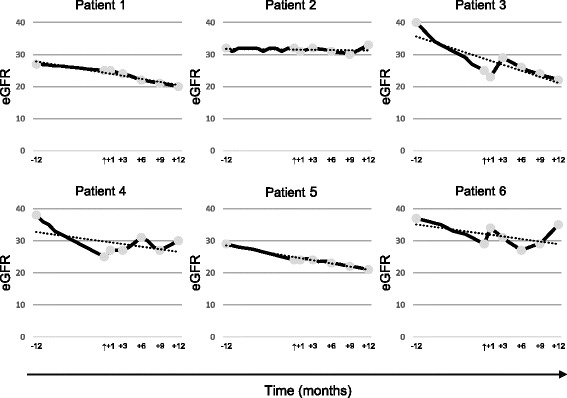
Estimated glomerular filtration rate (eGFR) changes in autosomal dominant polycystic kidney disease (ADPKD) patients from 1 year prior to mesenchymal stromal cell (MSC) infusion up to 12 months after the infusion

Patients had a mean eGFR value of 33.8 ± 5.3 ml/min/1.73 m^2^ 1 year before the cell infusion, which declined to 26.7 ± 3.1 ml/min/1.73 m^2^ at baseline (*P* = 0.03) and 25.8 ± 6.2 ml/min/1.73 m^2^ at the 12-month visit (*P* = 0.62). Figure 1 shows the patients’ eGFR changes. The mean SCr level of 2 ± 0.30 mg/dl within the same period increased to 2.5 ± 0.4 mg/dl at baseline (*P* = 0.04) and 2.5 ± 0.6 mg/dl at the 12-month follow-up (*P* = 0.96). There was a significant change between the differences of these two periods (-12, baseline and +12, baseline) in SCr (*P* = 0.05) but not in eGFR (*P* = 0.09; Table 4).

Systolic (*P* = 0.55) and diastolic (*P* = 0.43) BP did not significantly change 12 months after the cell infusion (Table 4). We observed no significant changes in renal function parameters and BP during the year after intervention (Additional file 1: Table S4).

#### Kidney length (KL)

There was a slight increase in the median left KL at baseline (18.3 ± 2.4 cm) to 12 months after the cell infusion (18.7 ± 2.6 cm; *P* = 0.06). The median right KL at baseline was 19.1 ± 2 cm which increased slightly to 19.5 ± 2 cm at 12 months (*P* = 0.13; Table 4).

## Discussion

To the best of our knowledge, this is the first clinical trial that has shown the safety and tolerability of a single BMMSC intravenous infusion in ADPKD patients.

However, the mean eGFR level noticeably decreased from 1 year before intervention to baseline (*P* = 0.03). We observed a slight, nonsignificant decrease for eGFR at 12-month follow-up that could not be considered exactly due to the intervention.

We observed enlargement of both kidneys in all patients at 12 months after the cell infusion that was probably due to usual cyst growth. We used KL to evaluate disease progression and, to some extent, represent the size of the kidney [20, 21]. However, KL is not as appropriate as HtTKV as proposed by the Consortium for Radiologic Imaging Studies of Polycystic Kidney Disease (CRISP) [22].

The HALT study showed a crucial role for hypertension (HTN) control in management of ADPKD [23]. Accordingly, we tightly controlled HTN in our patients prior to their enrollment. This did not change during the follow-up period.

Franchi et al. performed the only preclinical study that used allogeneic MSCs in a PKD rat model. They showed the beneficial effect of a single intravenous infusion for improvements in systolic HTN and fibrosis. Although the infusion did not affect cyst size and number, it had a significant improvement on cortical and parenchymal vasculature density, which resulted in better tubular function and improvements in creatinine clearance seen in the intervention group in the autosomal recessive polycystic kidney disease (ARPKD) model [13]. They also reported the safety of the MSC infusion with no mortality related to their protocol, which supported our safety results.

There is a growing body of clinical research which indicates that a systematic MSC infusion is safe under different conditions [24], including renal diseases [7, 9]. No AEs such as acute infusion toxicity, organ complications, infections, death or malignancies associated with systematic infusion of these cells have been reported in human studies [24]. In concordance with their results, our study showed the safety of an intravenous MSC infusion following comprehensive assessment of AEs, SAEs, and clinical and paraclinical parameters. There are also reports of potential efficacy and safety of MSC infusions in patients with different etiologies of CKD [9, 25–27]. However, there is a lack of data demonstrates the impact of MSCs on PKD.

ADPKD progression is associated with several pathways such as apoptosis [28], fibrosis [2, 29, 30], inflammation [2, 31, 32], cyst proliferation, renin–angiotensin system (RAS) activation [33, 34], and kidney vasculature impairment [13]. MSCs may affect these pathways through different mechanisms, as briefly discussed in the following.

Recent experimental studies reported that MSCs predominantly improved kidney tubular regeneration by secretion of growth factors, cytokines, and chemokines which decrease apoptosis, inflammation, and fibrosis [7], and subsequently ameliorate kidney function (creatinine clearance, eGFR, and HTN) and structure in CKD models [7, 9]. The potential capabilities of MSCs [35] are not limited to antifibrotic and anti-inflammatory effects on CKD models [11, 12] and its beneficial impact on apoptosis in an AKI model [10, 36] which could be useful for PKD.

Rapamycin decreases the proliferation of CD133+ and CD24+ cells and cyst formation in vitro through inhibition of the mechanistic target of rapamycin (mTOR) in PKD [17]. In our in vitro study, we observed that MSCs could reduce the proliferative potential and cyst formation of CD133+ progenitor cells from ADPKD patients (unpublished data).

The mitogen-activated protein kinase (MAPK) family is involved in proliferation, apoptosis, and fibrosis in PKD. MSC-derived conditioned medium has been shown to inhibit p38 mitogen-activated protein kinases (p38) and extracellular signal-regulated kinase (ERK) activity (MAPK family) as well as induce antiapoptotic effects in an AKI rat model [37].

Various studies illustrated that downregulation of pro-inflammatory cytokines reduced cyst formation and inflammation in ADPKD [38–40]. MSCs have the potential to decrease both the activity of the nuclear factor kappa B (NF-ĸB) pathway [11] and pro-inflammatory cytokines, which eventually lessen inflammation in a CKD model [12].

Following ADPKD progression, renovascular architecture disrupts where there is the vessel network around the cyst [13, 41]. Vascular endothelial growth factor (VEGF) is an antiapoptotic molecule [42] that can normalize the blood vessel network surrounding the cysts, BUN, SCr, and the cyst disorder as well as reduce macrophage pericystic infiltration in an ARPKD model [43]. VEGF C has been shown to decrease in an ARPKD mice model [43]. Human MSCs are a source of VEGF and may be useful for appropriate angiogenesis [44].

RAS inhibition improves BP and TKV, and prevents the spread of cyst formation in experimental animal models and humans [23, 45]. MSCs reduce renin, angiotensin-converting enzyme (ACE), and angiotensin II type 1 (AT1) receptor expression, and lead to reductions in BP, inflammation, and fibrosis [46, 47]. The MSC inhibitory effect on RAS is more stable than ACE inhibitors [47].

Taken together, following the safety and tolerability results of our study, the next step is to test the efficacy of MSC in ADPKD patients.

### Limitations

The current trial limitations included our inability to demonstrate the efficacy due to the study design that consisted of a small sample size, lack of a control group, short follow-up period, the single cell infusion, and not using htTKV as a surrogate endpoint.

## Conclusions

This trial demonstrated that an intravenous infusion of autologous MSCs was safe and well tolerated in ADPKD patients. We could not assess the efficacy due to the trial design. Our findings should be investigated in a randomized placebo-controlled trial with a larger population, which we intend to perform.

## Additional files

Additional file 1: Table S1. Method of kidney ultrasound imaging. DTPA kidney scan method. Cell parameters for the study patients. **Table S2.** Safety assessment of the mesenchymal stromal cell (MSC) infusion in 1 year following baseline visit of study patients. **Table S3.** Laboratory parameters at baseline, 1, 3, 6, 9, and 12 months of follow-up. **Table S4.** Changes in renal function parameters and blood pressure (BP) 1 year before, and at 1, 3, 6, 9, and 12 months after mesenchymal stromal cell (MSC) infusion. (DOCX 70 kb)

Additional file 2: Figure S1. Characterization of the patients’ bone mesenchymal stromal cells (MSCs). (DOCX 781 kb)

## Abbreviations

ACE: Angiotensin-converting enzyme
ADPKD: Autosomal dominant polycystic kidney disease
ADPLD: Autosomal dominant polycystic liver disease
AE: Adverse events
AKI: Acute kidney injury
ARPKD: Autosomal recessive polycystic kidney disease
AT1: Angiotensin II receptor type 1
BM: Bone marrow
BMA: Bone marrow aspiration
BMMSCs: Bone marrow mesenchymal stromal cells
BP: Blood pressure
BUN: Blood urea nitrogen
CKD: Chronic kidney disease
CRISP: Consortium for Radiologic Imaging Studies of Polycystic Kidney Disease
CTCAE: Common Terminology Criteria for Adverse Events
DSMB: Data Safety Monitoring Board
DTPA: Diethylenetriaminepentaacetic acid
eGFR: Estimated glomerular filtration rate
ERK: Extracellular signal-regulated kinase
ESRD: End-stage renal disease
FBS: Fetal bovine serum
FDO: Food and Drug Organization
FITC: Fluorescein isothiocyanate
GFR: Glomerular filtration rates
h: Hour
HLA: Human leukocyte antigen
HSC: Hematopoietic stem cell
HTN: Hypertension
htTKV: Height-adjusted total kidney volume
KL: Kidney length
LAL: Limulus amebocyte lysate
MAPK: Mitogen-activated protein kinase
MDRD: Modification of diet in renal disease
MRI: Magnetic resonance imaging
MSCs: Mesenchymal stromal cells
mTOR: Mechanistic target of rapamycin
NF-ĸB pathway: Nuclear factor kappa B - a prototypical pro-inflammatory signaling pathway
p38: p38 mitogen-activated protein kinases
PE: Phycoerythrin
PKD: Polycystic kidney disease
QCT: Quality control tests
RAS: Renin–angiotensin system
SAE: Serious adverse events
SCr: Serum creatinine
SD: Standard deviation
Tregs: Regulatory T cells
V2RA: Vasopressin II receptor antagonist
VEGF: Vascular endothelial growth factor
α-MEM: alpha modified Eagle medium

## References

[1] Iglesias CG, Torres VE, Offord KP, Holley KE, Beard CM, Kurland LT (1983). Epidemiology of adult polycystic kidney disease, Olmsted County, Minnesota: 1935-1980. Am J Kidney Dis.

[2] Torra R (2014). Autosomal dominant policystic kidney disease, more than a renal disease. Minerva Endocrinol.

[3] Chang MY, Ong AC (2013). New treatments for autosomal dominant polycystic kidney disease. Br J Clin Pharmacol.

[4] Bastos AP, Onuchic LF (2011). Molecular and cellular pathogenesis of autosomal dominant polycystic kidney disease. Braz J Med Biol Res.

[5] Torres VE, Chapman AB, Devuyst O, Gansevoort RT, Grantham JJ, Higashihara E, Perrone RD, Krasa HB, Ouyang J, Czerwiec FS (2012). Tolvaptan in patients with autosomal dominant polycystic kidney disease. N Engl J Med.

[6] Suzuki E, Fujita D, Takahashi M, Oba S, Nishimatsu H (2016). Adult stem cells as a tool for kidney regeneration. World J Nephrol.

[7] Morigi M, Rota C, Remuzzi G (2016). Mesenchymal stem cells in kidney repair. Methods Mol Biol..

[8] Dominici M, Le Blanc K, Mueller I, Slaper-Cortenbach I, Marini F, Krause D, Deans R, Keating A, Prockop D, Horwitz E (2006). Minimal criteria for defining multipotent mesenchymal stromal cells. The International Society for Cellular Therapy position statement. Cytotherapy.

[9] Hickson LJ, Eirin A, Lerman LO (2016). Challenges and opportunities for stem cell therapy in patients with chronic kidney disease. Kidney Int.

[10] Bruno S, Grange C, Collino F, Deregibus MC, Cantaluppi V, Biancone L, Tetta C, Camussi G (2012). Microvesicles derived from mesenchymal stem cells enhance survival in a lethal model of acute kidney injury. PLoS One.

[11] Wu HJ, Yiu WH, Li RX, Wong DW, Leung JC, Chan LY, Zhang Y, Lian Q, Lin M, Tse HF (2014). Mesenchymal stem cells modulate albumin-induced renal tubular inflammation and fibrosis. PLoS One.

[12] Semedo P, Correa-Costa M, Antonio Cenedeze M, Maria Avancini Costa Malheiros D, Antonia dos Reis M, Shimizu MH, Seguro AC, Pacheco-Silva A, Saraiva Camara NO (2009). Mesenchymal stem cells attenuate renal fibrosis through immune modulation and remodeling properties in a rat remnant kidney model. Stem Cells.

[13] Franchi F, Peterson KM, Xu R, Miller B, Psaltis PJ, Harris PC, Lerman LO, Rodriguez-Porcel M (2015). Mesenchymal stromal cells improve renovascular function in polycystic kidney disease. Cell Transplant.

[14] Moghadasali R, Mutsaers HA, Azarnia M, Aghdami N, Baharvand H, Torensma R, Wilmer MJ, Masereeuw R (2013). Mesenchymal stem cell-conditioned medium accelerates regeneration of human renal proximal tubule epithelial cells after gentamicin toxicity. Exp Toxicol Pathol.

[15] Moghadasali R, Azarnia M, Hajinasrollah M, Arghani H, Nassiri SM, Molazem M, Vosough A, Mohitmafi S, Najarasl M, Ajdari Z (2014). Intra-renal arterial injection of autologous bone marrow mesenchymal stromal cells ameliorates cisplatin-induced acute kidney injury in a rhesus Macaque mulatta monkey model. Cytotherapy.

[16] Moghadasali R, Hajinasrollah M, Argani H, Nassiri SM, Najarasl M, Sodeifi N, Baharvand H, Aghdami N (2015). Autologous transplantation of mesenchymal stromal cells tends to prevent progress of interstitial fibrosis in a rhesus Macaca mulatta monkey model of chronic kidney disease. Cytotherapy.

[17] Carvalhosa R, Deambrosis I, Carrera P, Pasquino C, Rigo F, Ferrari M, Lasaponara F, Ranghino A, Biancone L, Segoloni G (2011). Cystogenic potential of CD133+ progenitor cells of human polycystic kidneys. J Pathol.

[18] Pei Y, Obaji J, Dupuis A, Paterson AD, Magistroni R, Dicks E, Parfrey P, Cramer B, Coto E, Torra R (2009). Unified criteria for ultrasonographic diagnosis of ADPKD. J Am Soc Nephrol.

[19] Hartung EA (2016). Biomarkers and surrogate endpoints in kidney disease. Pediatr Nephrol.

[20] Vlajkovic S, Cukuranovic R, Bjelakovic MD, Ilic G, Jaksic T, Cukuranovic J (2010). Relative length of human kidney as more precise measuring of normal kidney. Med Pregl.

[21] Bhutani H, Smith V, Rahbari-Oskoui F, Mittal A, Grantham JJ, Torres VE, Mrug M, Bae KT, Wu Z, Ge Y (2015). A comparison of ultrasound and magnetic resonance imaging shows that kidney length predicts chronic kidney disease in autosomal dominant polycystic kidney disease. Kidney Int.

[22] Bae KT, Tao C, Wang J, Kaya D, Wu Z, Bae JT, Chapman AB, Torres VE, Grantham JJ, Mrug M (2013). Novel approach to estimate kidney and cyst volumes using mid-slice magnetic resonance images in polycystic kidney disease. Am J Nephrol.

[23] Schrier RW (2015). Blood pressure in early autosomal dominant polycystic kidney disease. N Engl J Med.

[24] Lalu MM, McIntyre L, Pugliese C, Fergusson D, Winston BW, Marshall JC, Granton J, Stewart DJ (2012). Canadian Critical Care Trials Group. Safety of cell therapy with mesenchymal stromal cells (SafeCell): a systematic review and meta-analysis of clinical trials. PLoS One.

[25] Tan J, Wu W, Xu X, Liao L, Zheng F, Messinger S, Sun X, Chen J, Yang S, Cai J (2012). Induction therapy with autologous mesenchymal stem cells in living-related kidney transplants: a randomized controlled trial. JAMA.

[26] El-Ansary M, Saadi G, Abd El-Hamid SM (2012). Mesenchymal stem cells are a rescue approach for recovery of deteriorating kidney function. Nephrology (Carlton).

[27] Lee H, Park JB, Lee S, Baek S, Kim H, Kim SJ (2013). Intra-osseous injection of donor mesenchymal stem cell (MSC) into the bone marrow in living donor kidney transplantation; a pilot study. J Transl Med..

[28] Riella C, Czarnecki PG, Steinman TI (2014). Therapeutic advances in the treatment of polycystic kidney disease. Nephron Clin Pract.

[29] Omori S, Hida M, Fujita H, Takahashi H, Tanimura S, Kohno M, Awazu M (2006). Extracellular signal-regulated kinase inhibition slows disease progression in mice with polycystic kidney disease. J Am Soc Nephrol.

[30] Sugiyama N, Kohno M, Yokoyama T (2012). Inhibition of the p38 MAPK pathway ameliorates renal fibrosis in an NPHP2 mouse model. Nephrol Dial Transplant.

[31] Nagao S, Yamaguchi T, Kusaka M, Maser RL, Takahashi H, Cowley BD, Grantham JJ (2003). Renal activation of extracellular signal-regulated kinase in rats with autosomal-dominant polycystic kidney disease. Kidney Int.

[32] Okumura Y, Sugiyama N, Tanimura S, Nishida M, Hamaoka K, Kohno M, Yokoyama T (2009). ERK regulates renal cell proliferation and renal cyst expansion in inv mutant mice. Acta Histochem Cytochem.

[33] Torres VE, Donovan KA, Scicli G, Holley KE, Thibodeau SN, Carretero OA, Inagami T, McAteer JA, Johnson CM (1992). Synthesis of renin by tubulocystic epithelium in autosomal-dominant polycystic kidney disease. Kidney Int.

[34] Saigusa T, Dang Y, Bunni MA, Amria MY, Steele SL, Fitzgibbon WR, Bell PD (2015). Activation of the intrarenal renin-angiotensin-system in murine polycystic kidney disease. Physiol Rep.

[35] Patel DM, Shah J, Srivastava AS (2013). Therapeutic potential of mesenchymal stem cells in regenerative medicine. Stem Cells Int..

[36] Zhou Y, Xu H, Xu W, Wang B, Wu H, Tao Y, Zhang B, Wang M, Mao F, Yan Y (2013). Exosomes released by human umbilical cord mesenchymal stem cells protect against cisplatin-induced renal oxidative stress and apoptosis in vivo and in vitro. Stem Cell Res Ther.

[37] Qi S, Wu D (2013). Bone marrow-derived mesenchymal stem cells protect against cisplatin-induced acute kidney injury in rats by inhibiting cell apoptosis. Int J Mol Med.

[38] Li X, Magenheimer BS, Xia S, Johnson T, Wallace DP, Calvet JP, Li R (2008). A tumor necrosis factor-alpha-mediated pathway promoting autosomal dominant polycystic kidney disease. Nat Med.

[39] Ta MH, Harris DC, Rangan GK (2013). Role of interstitial inflammation in the pathogenesis of polycystic kidney disease. Nephrology (Carlton).

[40] Lee EJ, Song SA, Mun HW, Yoo KH, Choi SY, Park EY, Park JH (2014). Blockade of interleukin-8 receptor signalling inhibits cyst development in vitro, via suppression of cell proliferation in autosomal polycystic kidney disease. Nephrology (Carlton).

[41] Xu R, Franchi F, Miller B, Crane JA, Peterson KM, Psaltis PJ, Harris PC, Lerman LO, Rodriguez-Porcel M (2013). Polycystic kidneys have decreased vascular density: a micro-CT study. Microcirculation.

[42] Ilic D, Almeida EA, Schlaepfer DD, Dazin P, Aizawa S, Damsky CH (1998). Extracellular matrix survival signals transduced by focal adhesion kinase suppress p53-mediated apoptosis. J Cell Biol.

[43] Huang JL, Woolf AS, Kolatsi-Joannou M, Baluk P, Sandford RN, Peters DJ, McDonald DM, Price KL, Winyard PJ, Long DA (2016). Vascular endothelial growth factor C for polycystic kidney diseases. J Am Soc Nephrol.

[44] Kagiwada H, Yashiki T, Ohshima A, Tadokoro M, Nagaya N, Ohgushi H (2008). Human mesenchymal stem cells as a stable source of VEGF-producing cells. J Tissue Eng Regen Med.

[45] Zafar I, Tao Y, Falk S, McFann K, Schrier RW, Edelstein CL (2007). Effect of statin and angiotensin-converting enzyme inhibition on structural and hemodynamic alterations in autosomal dominant polycystic kidney disease model. Am J Physiol Renal Physiol.

[46] Oliveira-Sales EB, Maquigussa E, Semedo P, Pereira LG, Ferreira VM, Camara NO, Bergamaschi CT, Campos RR, Boim MA (2013). Mesenchymal stem cells (MSC) prevented the progression of renovascular hypertension, improved renal function and architecture. PLoS One.

[47] Gregorini M, Corradetti V, Rocca C, Pattonieri EF, Valsania T, Milanesi S, Serpieri N, Bedino G, Esposito P, Libetta C (2016). Mesenchymal stromal cells prevent renal fibrosis in a rat model of unilateral ureteral obstruction by suppressing the renin-angiotensin system via HuR. PLoS One.

