# Supplementary material for: Safety and tolerability of autologous bone marrow mesenchymal stromal cells in ADPKD patients
Source: Stem Cell Res Ther. 2017 May 23;8:116. doi: 10.1186/s13287-017-0557-7 (PMC5442691; doi:10.1186/s13287-017-0557-7)
Supplement: Supplementary file 1 — Method of kidney ultrasound imaging. DTPA kidney scan method. Cell parameters for the study patients. Table S2. Safety assessment of the mesenchymal stromal cell (MSC) infusion in 1 year following baseline visit of study patients. Table S3. Laboratory parameters at baseline, 1, 3, 6, 9, and 12 months of follow-up. Table S4. Changes in renal function parameters and blood pressure (BP) 1 year before, and at 1, 3, 6, 9, and 12 months after mesenchymal stromal cell (MSC) infusion. (DOCX 70 kb) [file 13287_2017_557_MOESM1_ESM.docx]

**Supplementary 1**

**Kidney ultrasound imaging**

Patients underwent sonography with a real-time gray scale B-mode scanner and 3.5 MHz probes (RT-X200; GE Medical Systems, Milwaukee, WI). Although height-adjusted total kidney volume (htTKV) is considered a proper surrogate endpoint to assess the efficacy of intervention in autosomal dominant polycystic kidney disease (ADPKD) clinical trials, we have measured changes in kidney length (KL) by sonography. We measured KL (the longest pole to pole distance) in coronal or sagittal planes. All participants were requested to empty their bladders prior to the sonography procedure.

**DTPA kidney scan**

Glomerular filtration rate (GFR) was measured by renal dynamic imaging (modified Gate’s method) using 99mTc-diethylenetriaminepentaacetic acid (DTPA). Each patient was requested to drink 250-300 cc water 30 minutes before the procedure. We performed data acquisition according to the latest version of the Guidelines of the Society of Nuclear Medicine (SNM) by a single-headed Philips gamma camera. First, the syringe that contained 5-6 millicuries (85-222 MBqs) of a freshly prepared radiopharmaceutical was counted for 60 min at a 30-cm distance from the detector in order to register the pre-count measurement. Next, we placed each patient in a supine position and imaging was carried out in the posterior position. The study was stopped after 15 min and the above mentioned syringe was counted under the same conditions in order to determine the post-count measurement. Pre-count minus post-count values determined the injected dose. Regions of interest (ROIs) were drawn around the kidneys on the 1^st^-3^rd^ min images. Background ROIs were also drawn as semi-lunar areas inferolateral to the kidneys. We recorded each patient’s weight and height in the computer. GFR was calculated by a software program according to the modified Gate’s method by the following formula:

$$Total renal uptake percent (\%)=\frac{[(R-RB)/e^{\mu xR} +(L-LB)/e^{\mu xL}}{(Pre-Post)}$$

$$Global GFR=Total percent renal uptake \left( \% \right)\times100\times9.81270-6.82519$$

| Where:  Pre, pre-count; Post, post count; R, right kidney counts; RB, right kidney background counts; L, left kidney counts; LB, left kidney background counts; XR, right kidney depth; XL,: left kidney depth; µ,: attenuation coefficient of ^99m^Tc in soft tissue (0.153/cm); e, constant. |
| --- |

| **Table S1. Cell parameters for study patients.** | | | | | | |
| --- | --- | --- | --- | --- | --- | --- |
| Cell Parameters | Patient 1 | Patient 2 | Patient 3 | Patient 4 | Patient 5 | Patient 6 |
| Count (n) | 149×10^6^ | 95×10^6^ | 200×10^6^ | 100×10^6^ | 80×10^6^ | 110×10^6^ |
| Viability (%) | 99 | 92 | 98 | 90 | 96 | 94 |
| Markers (%) |  |  |  |  |  |  |
| CD90 | 99.7 | 94.2 | 95.1 | 98.1 | 97.2 | 98.1 |
| CD105 | 97.2 | 73.1 | 98.3 | 93.1 | 90 | 98.4 |
| CD73 | 99.1 | 91.9 | 96.3 | 98.5 | 91.7 | 89.5 |
| CD44 | 32.4 | 51.7 | 6.8 | 98.6 | 86.4 | 72.9 |
| CD11b | 98.7 | 4.4 | 5.1 | 0 | 33 | 17.2 |
| CD34 | 2.9 | 1.4 | 3.60 | 0.052 | 0.18 | 5.0 |
| CD45 | 0.5 | 1.1 | 0.5 | 0.0 | 0.1 | 0.6 |
| Microbial test BM | Negative | Negative | Negative | Negative | Negative | Negative |
| Microbial test MNC | Negative | Negative | Negative | Negative | Negative | Negative |
| Microbial test UM | Negative | Negative | Negative | Negative | Negative | Negative |
| Microbial test bulk | Negative | Negative | Negative | Negative | Negative | Negative |
| Microbial test final | Negative | Negative | Negative | Negative | Negative | Negative |
| Cytogenetic report | 46xy normal male karyotype | 46xx normal female karyotype | 46xy normal male karyotype | 46xy normal male karyotype | 46xx normal female karyotype | 46xx normal female karyotype |
| Mycoplasma test | Negative | Negative | Negative | Negative | Negative | Negative |
| LAL test (EU/ml) | < 0.125 | < 0.125 | < 0.125 | < 0.125 | < 0.125 | < 0.125 |
| Patient viral test |  |  |  |  |  |  |
| Anti-HCV Ab | Negative | Negative | Negative | Negative | Negative | Negative |
| Anti-HIV 1,2 | Non- reactive | Non- reactive | Non- reactive | Non- reactive | Non- reactive | Non- reactive |
| HBs Ag | Negative | Negative | Negative | Negative | Negative | Negative |
| Anti-HBc Ab | Negative | Negative | Negative | Negative | Negative | Negative |
| Anti-HBs Ab | Reactive | Non- reactive | Non- reactive | Non- reactive | Non- reactive | Non- reactive |
| Anti-HTLV | Negative | Negative | Negative | Negative | Negative | Negative |
| Cell count by neobar slide and nucleocounter; Viability test by nucleocounter and trypan blue; Cell markers by flow cytometry and analyzed by FlowJo software; Microbial tests by the BACTEC system, Mycoplasma by nested PCR; LAL by the endotoxin test; Viral tests were performed using ELISA.  BM bone marrow; MNC, mono nuclear cell; UM, upper medium. | | | | | | |

| **Table S2. Safety assessment of the mesenchymal stromal cell (MSC) infusion in one year following baseline visit of study patients.** | | | | | | |
| --- | --- | --- | --- | --- | --- | --- |
| Serious adverse event (SAE) | Pt 1 | Pt 2 | Pt 3 | Pt 4 | Pt 5 | Pt 6 |
| Death | n | n | n | n | n | n |
| Required hospitalization | n | n | y | n | n | n |
| ESRD | n | n | n | n | n | n |
| [Life-threatening](https://en.wikipedia.org/wiki/Death) | n | n | n | n | n | n |
| Any | n | n | n | n | n | n |
| Total | 0 | 0 | 1 | 0 | 0 | 0 |
|  |  | | | | | |
| Adverse event |  |  |  |  |  |  |
| Blood and lymphatic system disorders (y or n, grade) |  |  |  |  |  |  |
| Leukocytosis | n | n | n | n | n | n |
| Lymph node pain | n | n | n | n | n | n |
| Any | n | n | n | n | n | n |
| Cardiac disorders (y or n, grade) |  | | | | | |
| Acute coronary syndrome | n | n | n | n | n | n |
| Any | n | n | n | n | n | n |
| Ear and labyrinth disorders (y or n, grade) |  | | | | | |
| Ear pain | n | n | n | n | n | n |
| Vertigo | n | n | n | n | y,1 | n |
| Any | n | n | n | n | n | n |
| Endocrine disorders (y or n, grade) |  | | | | | |
| Hypothyroidism | n | n | n | n | n | n |
| Hyperparathyroidism | n | n | n | n | n | n |
| Any | n | n | n | n | n | n |
| Eye disorders (y or n, grade) |  | | | | | |
| Conjunctivitis | n | n | n | n | n | n |
| Dry eye | n | n | n | n | n | n |
| Any | n | n | n | n | n | n |
| Gastrointestinal disorders (y or n, grade) |  | | | | | |
| Bloating | n | n | n | n | n | n |
| Dyspepsia | n | n | n | n | n | y,2 |
| Diarrhea | n | n | y,1 | n | n | n |
| Nausea | n | y,1 | y,1 | y,1 | y,1 | y,1 |
| Any | n | n | n | n | n | n |
| General disorders and administration site conditions (y or n, grade) | | | | | | |
| Chills | n | n | n | n | y,1 | n |
| Fever | n | n | n | n | n | y,1 |
| Malaise | n | n | n | n | n | n |
| Infusion site reaction | n | n | n | n | n | n |
| Any | n | n | n | n | n | n |
| Hepatobiliary disorders (y or n, grade) |  | | | | | |
| Hepatic failure | n | n | n | n | n | n |
| Any | n | n | n | n | n | n |
| Immune system disorders (y or n, grade) |  | | | | | |
| Allergic reaction | n | n | n | n | n | n |
| Anaphylaxis | n | n | n | n | n | n |
| Any | n | n | n | n | n | n |
| Infections and infestations (y or n, grade) |  | | | | | |
| Bladder infection | n | n | n | n | n | n |
| Catheter related infection | n | n | n | n | n | n |
| Kidney infection | n | n | n | n | n | n |
| Hepatitis viral | n | n | n | n | n | n |
| Urinary tract infection | n | n | n | n | n | n |
| Any | n | n | n | n | n | n |
| Investigations (y or n, grade) |  | | | | | |
| Increased alanine aminotransferase | n | n | n | n | n | n |
| Increased alkaline phosphatase | n | n | n | n | n | n |
| Increased aspartate aminotransferase | n | n | n | n | n | n |
| Creatinine increased | n | n | y,1 | n | n | n |
| Hemoglobin increased | n | n | n | n | n | n |
| Lymphocyte count decreased | n | n | n | n | n | n |
| Lymphocyte count increased | n | n | n | n | n | n |
| Platelet count decreased | n | n | n | n | n | n |
| Platelet count decreased | n | n | n | n | n | n |
| Weight gain | n | n | n | n | n | n |
| Weight loss | y,1 | n | n | n | n | n |
| Any | n | n | n | n | n | n |
| Metabolism and nutrition disorders (y or n, grade) |  | | | | | |
| Hypercalcemia | n | n | n | n | n | n |
| Hyperkalemia | n | n | n | n | n | n |
| Hyponatremia | n | n | n | n | n | n |
| Hypokalemia | n | n | n | n | n | n |
| Hypoglycemia | n | n | n | n | n | n |
| Hypoalbuminemia | n | n | n | n | n | n |
| New-onset diabetes | n | n | n | n | n | n |
| Any | n | n | n | n | n | n |
| Musculoskeletal and connective tissue disorders (y or n, grade) | | | | | | |
| Arthritis | n | n | n | n | n | n |
| Arthralgia | n | n | n | n | n | n |
| Back pain | n | n | y,3 | n | n | n |
| Flank pain | n | n | n | y,1 | y,1 | n |
| Any | n | n | n | n | n | n |
| Neoplasms benign, malignant and unspecified include cysts and polyps (y or n, grade) | | | | | | |
| Benign | n | n | n | n | n | n |
| Malignant | n | n | n | n | n | n |
| Any | n | n | n | n | n | n |
| Nervous system disorders (y or n, grade) |  | | | | | |
| Cognitive disturbance | n | n | n | n | n | n |
| Dizziness | n | y,1 | y,1 | y,1 | y,1 | y,1 |
| Headache | y,2 | y,2 | y,2 | n | y,1 | n |
| Seizure | n | n | n | n | n | n |
| Any | n | n | n | n | n | n |
| Psychiatric disorders (y or n, grade) |  | | | | | |
| Anxiety | y,1 | n | n | n | n | y.1 |
| Confusion | n | n | n | n | n | n |
| Depression | n | n | n | n | n | n |
| Any | n | n | n | n | n | n |
| Renal and urinary disorders (y or n, grade) |  | | | | | |
| Acute kidney injury | n | n | y,1 | n | n | n |
| Chronic kidney disease | n | n | n | n | n | n |
| Proteinuria | y,1 | n | y.1 | n | y,1 | y,1 |
| Hematuria | n | n | n | n | n | n |
| Urinary frequency | n | n | n | n | n | n |
| Any | n | n | n | n | n | n |
| Reproductive system and breast disorders (y or n, grade) |  | | | | | |
| Breast pain | n | n | n | n | n | n |
| Any | n | n | n | n | n | n |
| Respiratory, thoracic, and mediastinal disorders (y or n, grade) | | | | | | |
| Allergic rhinitis | n | n | n | n | n | n |
| Cough | n | n | n | y,1 | n | y,1 |
| Dyspnea | n | n | n | n | n | n |
| Any | n | n | n | n | n | n |
| Skin and subcutaneous tissue disorders (y or n, grade) |  | | | | | |
| Pruritus | y,1 | y,1 | n | y,1 | y,1 | n |
| Eczema | n | n | n | n | n | n |
| Any | n | n | n | n | n | n |
| Vascular disorders (y or n, grade) |  | | | | | |
| Phlebitis | n | n | n | n | n | n |
| Any | n | n | n | n | n | n |
| Total | 5 | 4 | 8 | 5 | 8 | 7 |
| Pt, patient; y, yes; n, no. Adverse events were categorized according to the Common Terminology Criteria for Adverse Events (CTCAE) Version 4.0. | | | | | | |

| **Table S3. Laboratory parameters at Baseline, 1, 3, 6, 9 and 12 month follow-up.** | | | | | | |  |
| --- | --- | --- | --- | --- | --- | --- | --- |
| Patients Parameters | Baseline | 1 month | 3 months | 6 months | 9 months | 12 months | |
| Leukocytes (/ul) | 5700±885.4 | 6133.3±537.7 | 5733.3±372.4 | 5831.7±1026.8 | 6188.3±714.9 | 6468.3±2153.2 | |
| Hemoglobin (g/dl) | 12.8±1.7 | 12.8±1.6 | 13±1.2 | 12.9±1.2 | 12.8±1.3 | 13±1.2 | |
| HCT (%) | 37.4±3.8 | 38.4±4 | 38.9±2.1 | 38.8±3.5 | 38.9±4.4 | 39.5±3.4 | |
| MCV (fl) | 88.8±9.4 | 86.1±2.6 | 86.8±2.8 | 86±3.4 | 8.6±3.1 | 89.5±3.3 | |
| Platelets (*10^3^/ul) | 211.7±56.8 | 183.7±38.7 | 180±28.4 | 185.8±34.9 | 185.3±24.4 | 187.3±42 | |
| FBS (mg/dl) | 96.8±10 | 91.7±8.5 | 94±6.8 | 94.2±11.9 | 90.3±13.2 | 87.4±11.9 | |
| HbA1c (%) | 5.2±0.3 | NA | NA | NA | NA | 5.1±0.40 | |
| Sodium (mEq/l) | 141.8±1.9 | 141.3±2.1 | 141.5±1.8 | 141.5±3 | 141.6±4 | 141.3±2 | |
| Potassium (mEq/l) | 4.4±0.5 | 4.4±0.4 | 4.8±0.8 | 4.4±0.7 | 4.3±0.2 | 4.3±0.5 | |
| Calcium (mg/dl) | 9.3±0.6 | 9.5±0.8 | 9.2±0.4 | 9.4±0.4 | 9.8±0.5 | 9.4±0.3 | |
| Phosphorus (mg/dl) | 3.7±0.6 | 3.6±0.6 | 3.7±0.8 | 3.5±0.3 | 3.4±0.5 | 3.7±0.5 | |
| TSH (MIU/ml) | 2.3±1.1 | NA | NA | NA | NA | 2.4±1.3 | |
| PTH (pg/ml) | 140.3±71 | NA | NA | NA | NA | 112±48.3 | |
| ESR 1hr (mm/hr) | 22.2±18.8 | 15.4±6.7 | 11.8±9 | 10.8±10.5 | 14±11.9 | 15.5±10.3 | |
| CRP (mg/l) | 5.1±8.7 | NA | 2.7±4.7 | NA | NA | 0.3±0.9 | |
| Albumin (g/dl) | 4.3±0.3 | 4.2±0.2 | 4.5±0.4 | 4.3±0.2 | 4.4±0.4 | 4.4±0.5 | |
| Uric acid (mg/l) | 6.5±2.1 | 6.5±1.3 | 6.7±1.9 | 6.7±1.8 | 6.9±1.8 | 7.2±1.3 | |
| ALT(U/l) | 24.3±9.8 | 22±5.7 | 21.8±5.4 | 20.8±8.4 | 23.2±11.6 | 19.3±6.6 | |
| AST (U/l) | 29.7±18.5 | 21.8±6 | 24.8±2.9 | 19±3.8 | 25±19.1 | 20.5±5.8 | |
| Alkaline phosphatase (U/l) | 231.2±62.5 | 163.4±53.8 | 178.2±42.9 | 192±27.2 | 191.4±28.1 | 155.8±53.8 | |
| Total cholesterol (mg/dl) | 177±44.2 | 161.7±38.3 | 157.8±40.9 | 149±35.4 | 161±35.2 | 172.5±42 | |
| Triglycerides (mg/dl) | 191.7±150.7 | 183.7±106.7 | 162.7±63.1 | 172.5±58.5 | 174.3±72.5 | 192.5±108.2 | |
| LDL cholesterol (mg/dl) | 99.5±34 | 86.6±26.1 | 89.2±30.9 | 76.2±21.9 | 86.2±21.6 | 96±30.2 | |
| HDL cholesterol (mg/dl) | 39.2±11 | 42±12.8 | 41.5±13.3 | 38.7±10.5 | 38±10.6 | 41.3±12.3 | |
| Dipstick proteinuria ^a^ | 1.7±0.8 | 1.7±0.5 | 1.2±0.4 | 1.2±0.4 | 1.2±0.4 | 1.3±0.5 | |
| NA, not assessed; ul, microliter; g/dl, grams per deciliter; fl, femto liters; mEq/l, milli equivalents per liter; mg/dl, milligram per deciliter; MIU/ml, milli-international units per milliliter; pg/ml, pictogram per milliliter; mm/hr, millimeters per hour; mg/l, milligram per liter; U/lit, units per liter; a, negative or 1=0 mg/dl; 2=15-30 mg/dl; 3=30-100 mg/dl; 4=100-300 mg/d; 5=300-1000 mg/dl; 6 >1000 mg/dl | | | | | | |  |

| **Table S4. Changes in renal function parameters and blood pressure (BP) one year before, 1, 3, 6, 9, and 12 months after mesenchymal stromal cell (MSC) infusion.** | | | | | | | | | | | | | | |
| --- | --- | --- | --- | --- | --- | --- | --- | --- | --- | --- | --- | --- | --- | --- |
| Patient Parameters | -12 months | | | Baseline | | 1 month | | 3 months | | 6 months | | 9 months | | 12 months |
| Patient 1 | |  |  | |  | |  | |  | |  | |  | |
| eGFR | | 27 | 25 | | 25 | | 24 | | 22 | | 21 | | 20 | |
| GFR DTPA SCAN | | NA | 23 | | NA | | NA | | NA | | NA | | 15 | |
| SCr (mg/dl) | | 2.3 | 2.3 | | 2.2 | | 2.2 | | 2.4 | | 2.4 | | 2.6 | |
| Systolic BP (mm Hg) | | 133 | 135 | | 126 | | 124 | | 117 | | 119 | | 130 | |
| Diastolic BP (mm Hg) | | 75 | 80 | | 78 | | 78 | | 81 | | 72 | | 85 | |
| BUN (mg/dl) | | 16.5 | 20 | | 18.7 | | 20.5 | | 21.5 | | 25.1 | | 24.7 | |
| Dipstick proteinuria a | | NA | Trace | | Trace | | Neg | | Neg | | Neg | | Trace | |
| Patient 2 | |  |  | |  | |  | |  | |  | |  | |
| eGFR | | 32 | 32 | | 31 | | 32 | | 31 | | 33 | | 32 | |
| GFR DTPA SCAN | | NA | 41.6 | | NA | | NA | | NA | | NA | | 47.8 | |
| SCr (mg/dl) | | 2.3 | 2.3 | | 2.4 | | 2.3 | | 2.4 | | 2.2 | | 2.2 | |
| Systolic BP (mm Hg) | | 110/ | 115 | | 119 | | 122 | | 130 | | 127 | | 123 | |
| Diastolic BP (mm Hg) | | 80 | 79 | | 90 | | 85 | | 78 | | 81 | | 75 | |
| BUN (mg/dl) | | 39 | 31 | | 27 | | 22 | | 27 | | 28 | | 28 | |
| Dipstick proteinuria ^a^ | | NA | Neg | | Neg | | Neg | | Neg | | Neg | | Neg | |
| Patient 3 | |  |  | |  | |  | |  | |  | |  | |
| eGFR | | 40 | 25 | | 23 | | 26 | | 24 | | 22 | | 22 | |
| GFR DTPA SCAN | | NA | 24 | | NA | | NA | | NA | | NA | | NA | |
| SCr (mg/dl) | | 1.9 | 2.8 | | 3 | | 2.5 | | 2.7 | | 2.9 | | 3.1 | |
| Systolic BP (mm Hg) | | 120 | 110 | | 133 | | 124 | | 121 | | 125 | | 117 | |
| Diastolic BP (mm Hg) | | 73 | 70 | | 81 | | 78 | | /68 | | 72 | | 79 | |
| BUN (mg/dl) | | 37 | 16.8 | | 25.7 | | 25.2 | | 26.5 | | 33.2 | | 21.2 | |
| Dipstick proteinuria ^a^ | | NA | Trace | | Trace | | Neg | | Neg | | Neg | | Neg | |
| Patient 4 | |  |  | |  | |  | |  | |  | |  | |
| eGFR | | 38 | 25 | | 27 | | 27 | | 31 | | 27 | | 25 | |
| GFR DTPA SCAN | | NA | 28 | | NA | | NA | | NA | | NA | | NA | |
| SCr (mg/dl) | | 2.1 | 3 | | 2.8 | | 2.8 | | 2.5 | | 2.8 | | 3 | |
| Systolic BP (mm Hg) | | 133 | 131 | | 133 | | 130 | | 129 | | 126 | | 131 | |
| Diastolic BP (mm Hg) | | 76 | 70 | | 75 | | 75 | | 80 | | 78 | | 78 | |
| BUN (mg/dl) | | 24 | 34.5 | | 33 | | 30 | | 39 | | 34 | | 36 | |
| Dipstick proteinuria ^a^ | | NA | Neg | | Neg | | Neg | | Neg | | Neg | | Neg | |
| Patient 5 | |  |  | |  | |  | |  | |  | |  | |
| eGFR | | 29 | 24 | | 24 | | 23 | | 23 | | 22 | | 21 | |
| GFR DTPA SCAN | | NA | 26 | | NA | | NA | | NA | | NA | | NA | |
| SCr (mg/dl) | | 1.87 | 2.5 | | 2.2 | | 2.27 | | 2.2 | | 2.3 | | 2.4 | |
| Systolic BP (mm Hg) | | 100 | 133 | | 131 | | 130 | | 128 | | 129 | | 131 | |
| Diastolic BP (mm Hg) | | 80 | 73 | | 76 | | 78 | | 77 | | 70 | | 78 | |
| BUN (mg/dl) | | 30 | 44 | | 33 | | 46 | | 41 | | 42 | | 40 | |
| Dipstick proteinuria ^a^ | | NA | Neg | | Trace | | Neg | | Trace | | Trace | | Trace | |
| Patient 6 | |  |  | |  | |  | |  | |  | |  | |
| eGFR | | 37 | 29 | | 34 | | 31 | | 27 | | 29 | | 35 | |
| GFR DTPA SCAN | | NA | 32.83 | | NA | | NA | | NA | | NA | | NA | |
| SCr (mg/dl) | | 1.5 | 1.9 | | 1.6 | | 1.7 | | 1.9 | | 1.8 | | 1.5 | |
| Systolic BP (mm Hg) | | 117 | 127 | | 135 | | 131 | | 128 | | 130 | | 129 | |
| Diastolic BP (mm Hg) | | 82 | 83 | | 80 | | 84 | | 78 | | 84 | | 78 | |
| BUN (mg/dl) | | 24.3 | 20.5 | | 27.5 | | 30.3 | | 23.3 | | 22.9 | | 27.1 | |
| Dipstick proteinuria ^a^ | | NA | + | | Trace | | Trace | | Neg | | Neg | | Neg | |
| BMI, body mass index; eGFR, estimated glomerular filtration rate; BP, blood pressure; SCr, serum creatinine; BUN, blood urea nitrogen; NA, not assessed. ^a^ Dipstick proteinuria range is defined as follow: Neg: 0 mg/dl, trace:15-30 mg/dl, +:30-100 mg/dl, ++:100-300 mg/d, +++: 300-1000 mg/dl, ++++: >1000 mg/dl. | | | | | | | | | | | | | | |
