# Supplementary figures and images for: Safety and tolerability of autologous bone marrow mesenchymal stromal cells in ADPKD patients
Source: Stem Cell Res Ther. 2017 May 23;8:116. doi: 10.1186/s13287-017-0557-7 (PMC5442691; doi:10.1186/s13287-017-0557-7)

Figure S1.


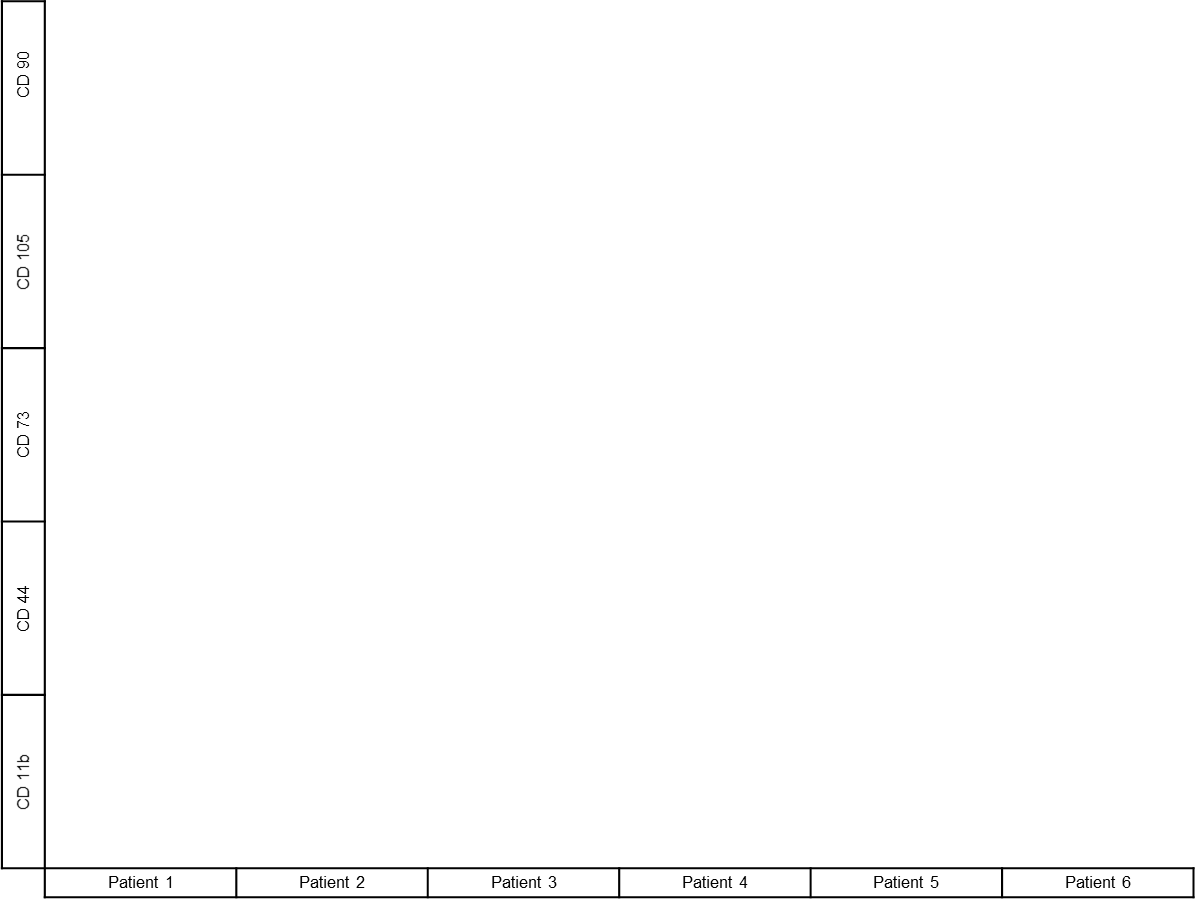


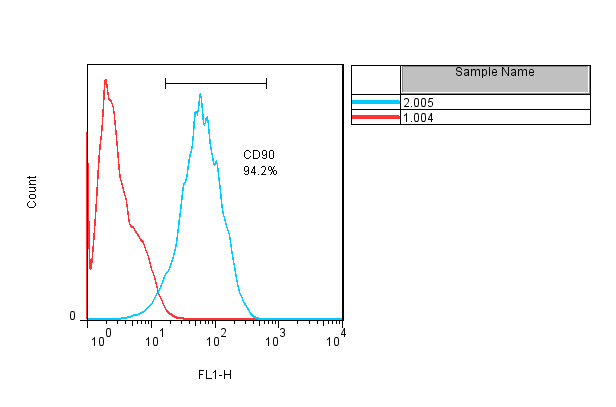

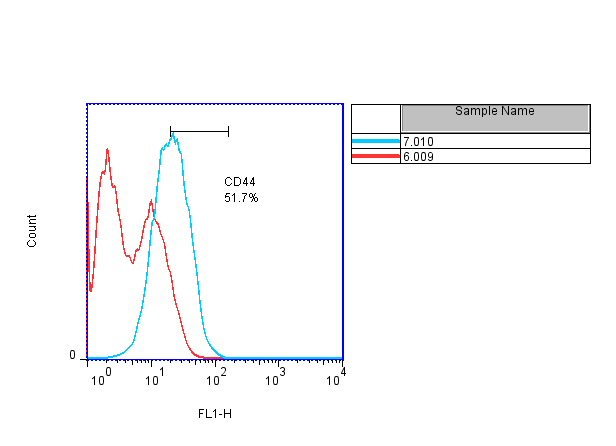

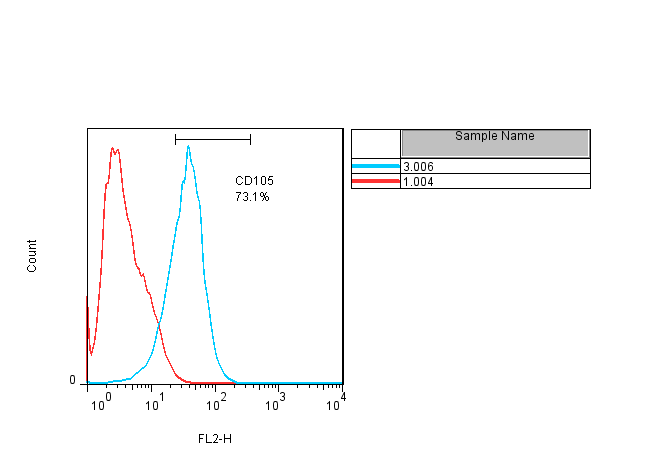

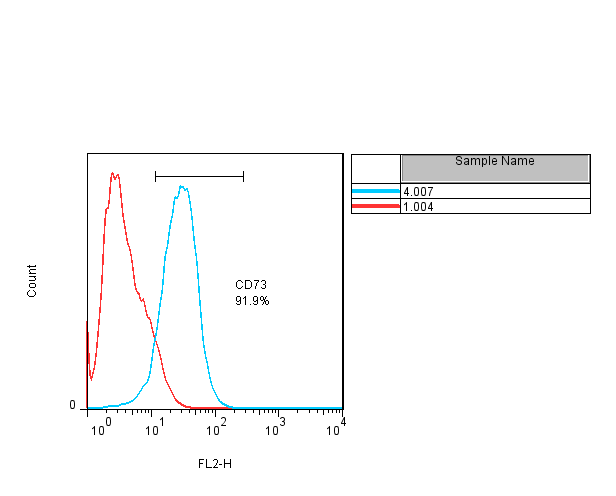

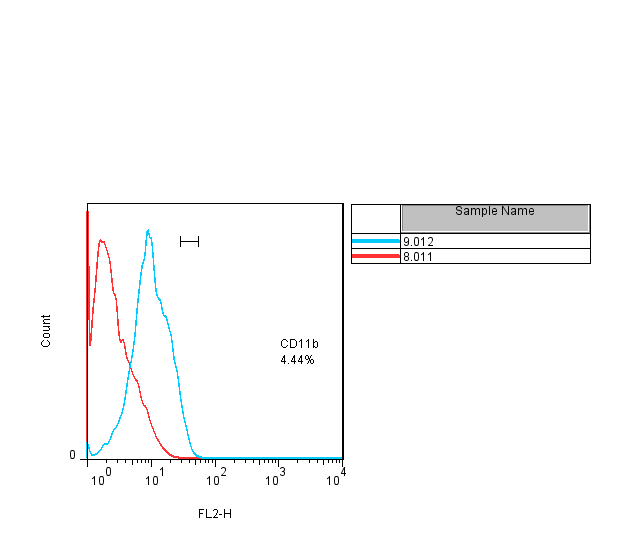

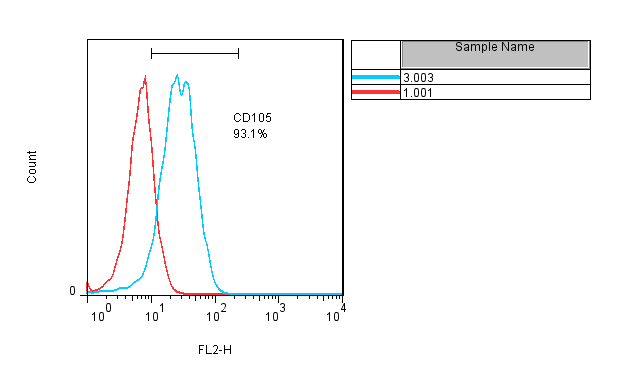

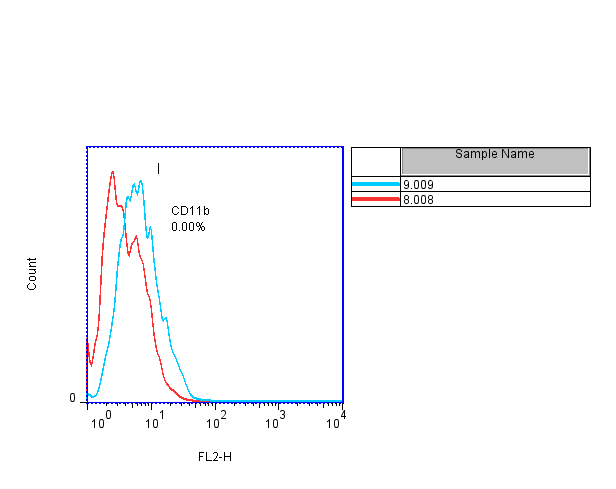

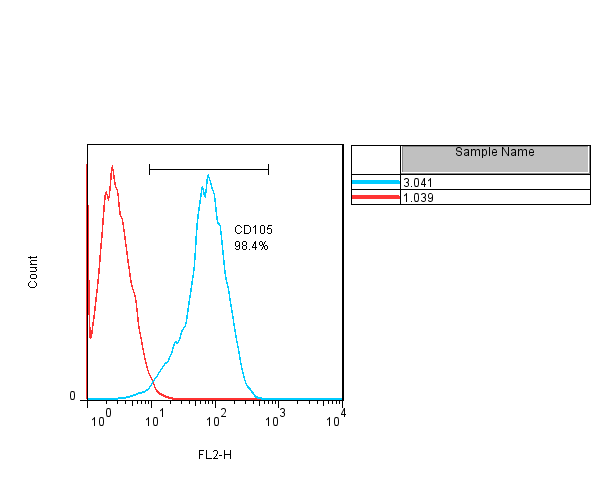

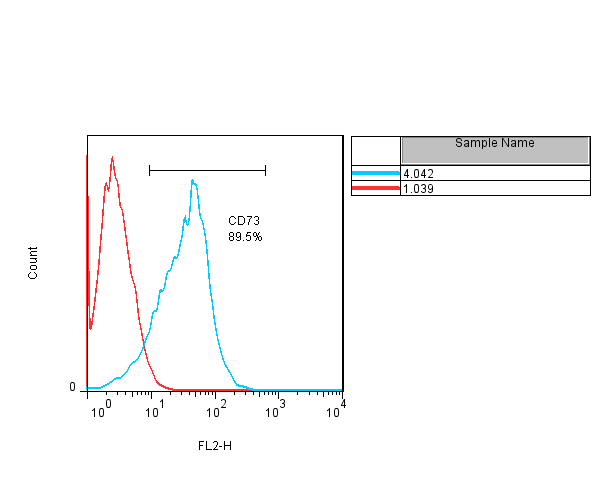

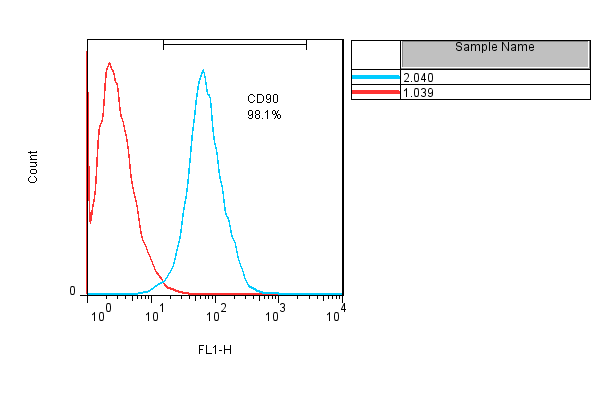

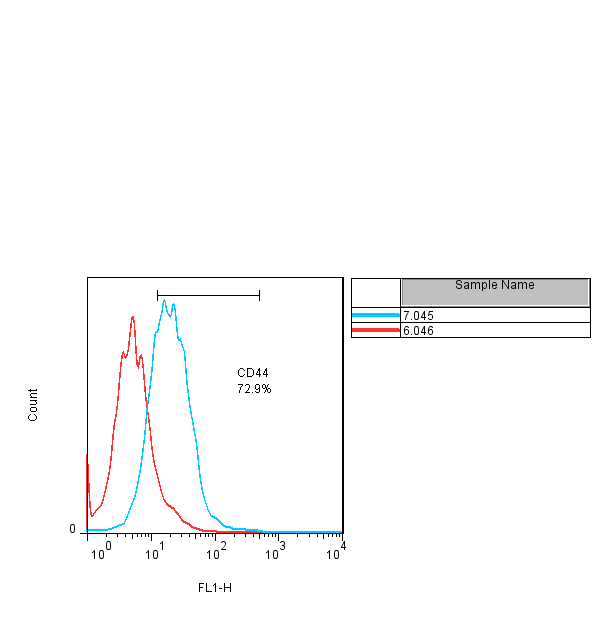

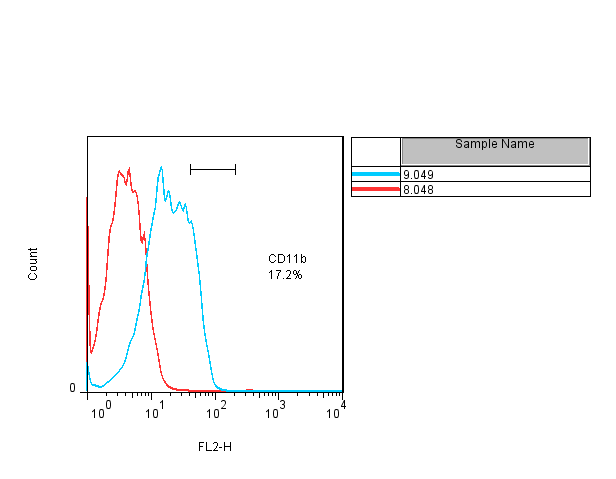


99.7 %

97.2 %

99.1 %

32.4 %

98.7 %

94.2 %

73.1 %

91.9 %

51.7 %

4.44 %

95.1 %

98.3 %

96.3 %

6.8 %

5.1 %

98.1 %

93.1 %

98.5 %

98.6 %

0 %

97.2 %

90.0 %

91.7 %

86.4 %

33.0 %

98.1 %

98.4 %

89.5 %

72.9 %

17.2 %

Supplement: Supplementary file 2 — Characterization of the patients’ bone mesenchymal stromal cells (MSCs). (DOCX 781 kb) [file 13287_2017_557_MOESM2_ESM.docx]
